# Person-to-Person Household and Nosocomial Transmission of Andes Hantavirus, Southern Chile, 2011

**DOI:** 10.3201/eid2010.140353

**Published:** 2014-10

**Authors:** Constanza Martinez-Valdebenito, Mario Calvo, Cecilia Vial, Rita Mansilla, Claudia Marco, R. Eduardo Palma, Pablo A. Vial, Francisca Valdivieso, Gregory Mertz, Marcela Ferrés

**Affiliations:** Pontificia Universidad Católica de Chile, Santiago, Chile (C. Martinez-Valdebenito, R.E. Palma, M. Ferrés);; Universidad Austral de Chile, Valdivia, Chile (M. Calvo);; Clínica Alemana–Universidad del Desarrollo, Santiago, Chile (C. Vial, C. Marco, P.A. Vial, F. Valdivieso);; Secretaría Regional Ministerial, Valdivia, Chile (R. Mansilla);; University of New Mexico, Albuquerque, New Mexico, USA (G. Mertz)

**Keywords:** hantavirus, hantavirus infections, nosocomial, viruses, household, Chile, transmission, person-to-person, Andes hantavirus, ANDV

## Abstract

Andes hantavirus (ANDV) causes hantavirus cardiopulmonary syndrome in Chile and is the only hantavirus for which person-to-person transmission has been proven. We describe an outbreak of 5 human cases of ANDV infection in which symptoms developed in 2 household contacts and 2 health care workers after exposure to the index case-patient. Results of an epidemiologic investigation and sequence analysis of the virus isolates support person-to-person transmission of ANDV for the 4 secondary case-patients, including nosocomial transmission for the 2 health care workers. Health care personnel who have direct contact with ANDV case-patients or their body fluids should take precautions to prevent transmission of the virus. In addition, because the incubation period of ANDV after environmental exposure is longer than that for person-to-person exposure, all persons exposed to a confirmed ANDV case-patient or with possible environmental exposure to the virus should be monitored for 42 days for clinical symptoms.

## Abstract 2

Four persons became ill after exposure to a patient infected with the virus; 2 cases involved hospital transmission.

Hantavirus cardiopulmonary syndrome (HCPS) is caused by infection with New World hantaviruses. First described in 1993 in the southwestern United States, HCPS has been documented throughout the Americas (*1*,*2*). For human cases, the mean incubation period of hantavirus infection from exposure to illness onset is 18.5 (range 7–42) days (*3*). As of December 31, 2013, a total of 848 human HCPS cases had been reported in Chile; the case-fatality rate has ranged from 32% to 35% per year (*4*).

The sole confirmed etiologic agent of HCPS in Chile is Andes virus (ANDV). Human infection with this virus occurs from exposure to contaminated excreta and secretions of rodents of the family *Cricetidae*. Transmission of ANDV between rodents has been experimentally documented after exposure of seronegative rodents to inhalation of aerosolized infected rodent secretions (*5*). ANDV is endemic in Chile and Argentina and is the only hantavirus for which person-to-person transmission has been documented. Person-to-person transmission of ANDV occurs mainly in family clusters or, less commonly, after activities in which close contact with an infected case-patient has occurred, primarily during the disease prodrome (*6*–*8*). A prospective study in Chile found that sexual partners and other close household contacts of ANDV-infected persons showed a 10-fold higher risk of acquiring the virus than household contacts who did not share bed or bedroom with the index case-patient (*3*,*9*).

Nosocomial transmission of ANDV has been a matter of concern for infection control practice and for health care workers who provide care for these patients, and in particular for workers who perform invasive procedures. In Argentina, person-to-person transmission of ANDV was documented in a physician who acquired infection after exposure to an ANDV-infected patient shortly after onset of the febrile prodrome (*7*,*8*). Although person-to-person transmission in Chile has been epidemiologically documented (*10*), nosocomial transmission has not been reported. Seroprevalence studies conducted among health care workers in hospitals in Chile where patients with ANDV infection have been treated have reported that health care workers exhibited ANDV IgG antibody at a proportion similar to that of the general population (*11*,*12*). Similarly, a study in the southwestern United States found no evidence of nosocomial transmission of another hantavirus, Sin Nombre virus (*13*).

We describe an outbreak of 5 cases of ANDV infection that occurred in a small, rural community in southern Chile in 2011. We present the epidemiologic and the clinical features of the cases, along with the molecular analysis of the virus strains from each case. Epidemiologic and virus sequence analyses support person-to-person transmission of ANDV in 4 of these cases, including nosocomial transmission in 2 cases.

## Materials and Methods

### Study Population

A case cluster of 5 human case-patients, including 2 persons involved in health care, occurred in Corral, Los Rios, Chile, during February–April 2011. Clinical history and information from epidemiologic questionnaires were obtained for each patient; all 5 had an acute febrile illness and signs and symptoms compatible with hantavirus infection. Acute infection was confirmed by detection of IgM against viral nucleoprotein antigen and real-time reverse transcription PCR (RT-PCR) targeting the small RNA segment of ANDV in blood samples obtained from these patients during the acute illness (*14*,*15*). Samples from 7 additional patients who had had HCPS in the same geographic region in previous years were used as controls for virus sequence analysis. All participants signed an informed consent approved by an ethics committee.

### Geographic and Demographic Features of Corral

Corral is a coastal town (39°52′0″ S, 73°25′60″ W) located 15 km west of Valdivia, the capital of the Los Rios region in Chile; the town is in the foothills of a coastal mountain range in the Valdivian rainforest ecoregion (*16*). The population is ≈5,433 inhabitants. Corral has 1 primary care hospital with 5 beds (hospital I); all patients with complications are transferred to a regional care center in Valdivia that has intensive care facilities (hospital II). Since 1997, a total of 13 cases of hantavirus infection have been reported in Corral, including the 5 cases described in this report (*17*). Prior to this report, the last 2 confirmed cases were in 2008 and 2010.

### Outbreak Description

On March 20, 2011, two suspected cases of hantavirus infection were reported. The patients were a 31-year-old woman (case-patient B) who worked as a nursing assistant at hospital I and a 53-year-old woman (case-patient C). Both lived near Corral. In addition to these cases, in late February, a 73-year-old man (case-patient A), the spouse of case-patient C, had been transferred from hospital I to hospital II for treatment of a pulmonary disease and evaluated for hantavirus infection; initial serologic testing results at a national reference laboratory were negative. On March 22, a fourth patient (case-patient D), a 60-year-old female housekeeper at hospital I, was admitted to hospital II with respiratory failure; she died a few hours later. A fifth patient (case-patient E), a 34-year-old man who was the husband of case-patient B, was hospitalized on April 3 at hospital II. On April 3, an epidemiologic investigation was initiated by the Health and Epidemiology Service, including investigation of infection control measures used at hospital I. 

### Genetic Characterization of the Virus

RNA was obtained from patients’ leukocytes from diagnostic samples and extracted by using the High Pure Viral RNA Kit (Roche Diagnostic GmbH, Roche Applied Science, Mannheim, Germany), according to the manufacturer’s instructions. For segment amplification, heminested RT-PCR was performed as described previously (*16*). Two portions of the medium segment, Gn and Gc, were amplified (Table 1), and the amplicons underwent agarose gel purification and sequencing in both directions. The chromatogram of each sequence was analyzed and aligned to generate a consensus sequence by using BioEdit version 7.1.11 (http://www.mbio.ncsu.edu/bioedit/bioedit.html). Twelve sequences were aligned by using ClustalW (http://www.clustal.org). Sequences were phylogenetically analyzed by conducting maximum-likelihood (ML) and Bayesian methodology on the concatenated Gn and Gc sequences. For ML, PAUP* version 4.0 (*18*) was used for a heuristic search with 100 random additions and branch swapping via tree-bisection-reconnection (*19*). jModeltest 3.7 was used to choose the best-fitting model of sequence evolution (*20*). The corrected Akaike information criterion (Akaike 1974) identified the Kimura 81 unequal base frequencies + gamma model (K81uf + Γ) as optimal (−lnL = 1251.2770, Akaike information criterion = 2515.7539, G = 1.5780), with base frequencies A = 0.2868, C = 0.3132, G = 0.0670, and T = 0.3329. Reliability of nodes in the ML tree was estimated by bootstrap analysis (*21*) obtained after 1,000 pseudo-replicates. The tree was rooted on the basis of the outgroup criterion by using the ANDV sequence available in GenBank (accession no. NC_003467.2). Sequences also were analyzed in a Bayesian framework to estimate the posterior probabilities of phylogenetic trees. Ten million phylogenetic trees were generated; the first 1,000 trees of the sample were removed to avoid including trees before convergence of the Markov Chain. As 2 independent molecular markers were used, a general likelihood–based mixture model of sequence evolution was applied as described (*22*). This model accommodates cases in which different sites in the alignment evolved in qualitatively distinct ways but does not require prior knowledge of these patterns or partitioning data. These analyses were conducted by using Bayes Phylogenies software (*22*). To find the best mixture model of evolution, the number of general time reversible matrices was estimated by using a reversible-jump Markov chain Monte Carlo method (*23*).

**Table 1:** Primers used for M segment amplification and sequencing of Andes hantavirus

Primer identification	Primer sequences, 5′ → 3′
GN1+	TAGTAGTAGACTCCGCAAGAAGAAG
GN534−	TCCTGCTKKTAAACACACTAGCCAT
GC94+	TGCAAATGATTGTGTTAGTAACACCA
GC674−	GTATTAGAGCCCCTAGCACAGGTT

## Results

### Laboratory and Epidemiologic Investigations

**Figure 1:**
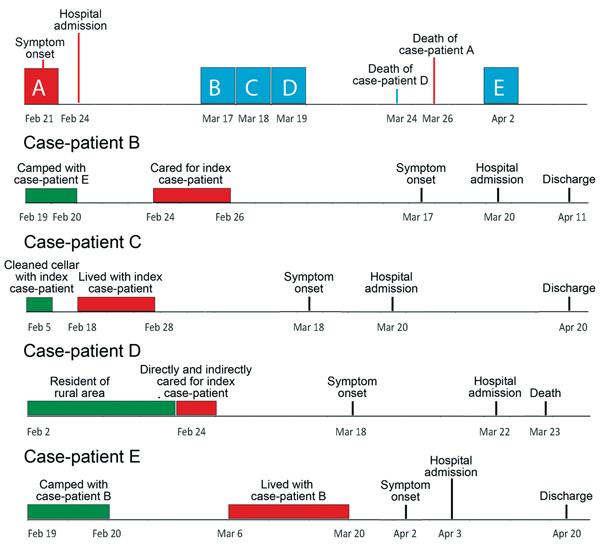
Timelines showing progression and key events related to each case-patient (A–E) in a cluster of 5 Andes hantavirus cases, southern Chile, 2011. Blue boxes along timeline for index case-patient (A) indicate date of illness onset for subsequent case-patients; green boxes indicate environmental exposures (exposure for case-patient A was the same as for case-patient C); red boxes indicate contact with other case-patients.

IgM and IgG against ANDV were detected in serum samples, and ANDV RNA was detected by RT-PCR in blood for all 5 patients in the cluster (Table 2; Figure 1). Case-patient A, the 73-year-old man, was identified as the index case-patient of the cluster. 

**Table 2:** Clinical and epidemiologic features of 5 patients involved in outbreak of ANDV infection, Chile, 2011*

**Feature**	**Case-patient A†**	**Case-patient B**	**Case-patient C**	**Case-patient D**	**Case-patient E**
**Age, y/sex**	73/M	31/F	53/F	60/F	34/M
**Occupation**	Farmer	Nursing assistant at hospital	Teacher	Cleaning personnel at hospital	Car mechanic
**Relationship to other case-patients**	Husband of case-patient C	Health care provider for case-patient A	Wife of case-patient A	Health care assistant for case-patient A	Husband of case-patient B
**Date of symptom onset**	Feb 21	Mar 17	Mar 18	Mar 18	Apr 2
**Date of hospitalization**	Feb 24	Mar 20	Mar 20	Mar 22	Apr 3
**Signs and symptoms**					
** Fever**	Yes	Yes	Yes	Yes	Yes
** Respiratory symptoms‡**	Yes	Yes	No	Yes	Yes
** Gastrointestinal symptoms§**	No	No	Yes	Yes	No
** Other symptoms¶**	No	Yes	Yes	Yes	Yes
**Mechanical ventilation, d**	28	8	0	1	6
**Hospitalization, d**	30	22	12	1	17
**Outcome**	Died	Survived	Survived	Died	Survived
**Days from environmental exposure to onset of symptoms**	16	25–26	41	7–45	41–42
**Days from exposure to hantavirus case-patient to onset of symptoms**	NA	19–21	22–25	18–20	13–27
**Laboratory test results on admission**					
** Platelet count, × 10^3^/μL**	51	56	108	101	147
** Leukocytes, × 10^3^ cells/μL**	4,67	5,46	1,21	3,92	11,46
** Hematocrit, %**	52	39	39	45	44
** Lymphocytes, %**	12	7	39	19	7
** Immunoblasts, %**	Yes	Yes	Yes	NR	Yes
** IgM/IgG for ANDV**	Negative#	Positive	Positive	Positive	Positive
** RT-PCR ANDV in blood cells**	ND	Positive	Positive	Positive	Positive

*ANDV, Andes virus; NA, not applicable; NR, not reported; RT-PCR, reverse transcription PCR; ND, not done. †Index case-patient. ‡Dry cough, dyspnea, cyanosis, crepitus. §Vomiting, diarrhea, nausea. ¶Severe headache, meningeal signs, myalgia, arthralgia, conjunctival infection, chills, photophobia, facial edema. **#On hospital admission. Repeat testing after 24 days yielded positive results.**

Case-patient A lived in a small settlement near Corral. His main risk activity was the cleaning of a home cellar where he was moving tiles on February 5. The cellar was heavily contaminated with rodent feces. The patient was admitted to hospital I on February 24 after 3 days of fever, dry cough, weakness, and progressive dyspnea. During hospitalization, he experienced progressive respiratory compromise, productive cough, and intense sweating that required frequent changes of gowns, sheets, and blankets. On February 26, he was transferred to the critical care unit at hospital II for mechanical ventilation. Serum samples were sent to the National Reference Laboratory 11 days after onset of his symptoms; results were negative for ANDV IgM. When the epidemiologically related hantavirus case-patients were admitted to hospital II, ANDV IgM testing was repeated, 24 days after onset of his symptoms, and results were positive. Case-patient A died on March 26 after 28 days of mechanical ventilation and use of vasoactive drugs.

Case-patient B, a nursing assistant at hospital I, exhibited a fever on March 17. She was hospitalized on March 20 and the same day was transferred from hospital I to the intensive care unit at hospital II. Severe shock and respiratory failure developed, and high doses of vasopressors and mechanical ventilation were required. A diagnosis of HCPS caused by ANDV infection was confirmed after 8 days of symptoms, and she was discharged on April 11. This patient had direct contact with case-patient A at hospital I from February 24–26, during his febrile prodrome and progression to the cardiopulmonary phase. She changed the patient’s clothes, sheets, and blankets because he perspired profusely. In addition, having met the patient previously, she greeted case-patient A with a kiss on his cheek several times during his hospitalization. She also had close contact with her husband at their home from the time she cared for the index case-patient through the first 3 days of her illness. She recalled possible environmental exposure from camping at 2 local beaches during February 1–4 and February 19–20; she collected wood and cleaned the area to set up tents.

Case-patient C was the spouse of case-patient A. She shared the same bed and cared for him during his febrile prodrome but denied that they had sexual activity after symptom onset. She entered the contaminated cellar with her husband but did not participate actively in his work in this area. On March 18, twenty-five days after her husband’s illness onset and 41 days after they entered the cellar, she exhibited mild fever, severe headache, myalgia, and photophobia . She sought medical attention at hospital II while her husband was still hospitalized, and acute ANDV infection was confirmed on March 24. Her chest radiograph results were normal. Her most remarkable symptoms were headache and irritability, and she had meningeal signs. Testing of cerebrospinal fluid (CSF) showed 8 white mononuclear cells, normal glucose levels, and a slightly elevated protein level of 0.5 g/L. CSF testing by RT-PCR for ANDV and ELISA for ANDV-specific IgG yielded negative results.

Case-patient D, a housekeeper at hospital I, had fever, abdominal pain, and vomiting develop on March 18. Two days later, she was hospitalized at hospital I, and 4 days later, she was transferred to hospital II, where severe shock and respiratory failure developed. She died a few hours after admission to hospital II. Her diagnosis was confirmed by positive results of serologic testing and RT-PCR for ANDV. She had direct and indirect contact with case-patient A while he was at hospital I. She entered his room and helped the nursing assistant (case-patient B) change his clothes and remove his sheets and bedclothes for washing.

Case-patient E, the husband of case-patient B, had fever, headache, myalgia, and back pain develop on April 2, and he was admitted to hospital II on April 3. Serologic testing for ANDV IgM and IgG after 5 days of symptoms yielded negative results, but results of RT-PCR for ANDV RNA were positive. IgM and IgG seroconversion were confirmed 10 days after symptom onset. The person-to-person exposure period for this patient was March 6–20; his possible environmental exposure exceeded the known incubation period for ANDV (*11*). Shock and respiratory failure developed, and he required mechanical ventilation and vasopressors but survived.

### Environmental Investigation

Rodent trapping was performed for 2 and 3 nights, respectively, at the 2 sites where case-patients reported possible environmental exposure: the cellar of the home of case-patients A and C and a camping area used by case-patients B and E (Table 3). Rodent serum samples were tested for ANDV antibodies by strip immunoblot assay (*24*); results were positive for 1 *Abrothrix longipilis* rodent trapped at the camping site. However, RT-PCR results for this sample were negative, and testing of rodents trapped at the home of case-patients A and C yielded negative results.

**Table 3:** Results of environmental investigation for 4 cases of ANDV infection, Chile, 2011*

Case-patients	Days after case-patient diagnosis	No. trapping nights	No. trapped rodents	No. traps per night	Rodent species trapped	SIA results, n = 24	RT-PCR results
B and E	63	3	46	57, 40, 40	*Abrothrix longipilis, A. olivaceus*, other *Abrothrix* sp., *Oligoryzomys longicaudauts*	1 positive (*A. longipilis*)	Negative
A and C	90	2	9	68, 68	*A. olivaceus, O. longicaudauts, Rattus norvegicus, R. rattus*	Negative	ND

*SIA, strip immune assay; RT-PCR, reverse transcription PCR; ND, not done.

### Viral Molecular Analysis

A portion of 942 bp of the ANDV small RNA segment was amplified and sequenced from samples of each of the 5 patients in the case cluster. Sequences aligned by using ClustalW showed 100% identity (data not shown), an observation consistent with the high degree of conservation of the small segment among hantaviruses (*7*,*25*).

Virus variability was established by comparing a portion of 914 bp of the highly variable ANDV medium RNA segment. The sequences obtained for the 2 medium segments encoding the ANDV glycoproteins Gn and Gc were compared separately (data not shown) and concatenated. Results were visualized in the identity matrix of concatenated sequences and showed that the concatenated sequences derived from the 5 cases in the cluster were similar to each other but differed from viral sequences from 7 patients who acquired ANDV in the same community in previous years (Table 4). The molecular identity of the concatenated Gn and Gc sequences between cases ranged from 99% to 100%, whereas the comparison with control sequences from the same geographic region ranged from 97% to 99%. These values show higher identity between the sequences derived from the cluster cases compared with other human cases from the same geographic region from previous years. All sequences obtained in this study have been deposited in GenBank (accession nos. KC567258–KC567281).

**Table 4:** Identity matrix of concatenated Gn and Gc sequences of ANDV isolates from the 5 case-patients in this study compared with sequences from ANDV samples from previous case-patients in the same geographic region of Chile*

**Sequence**	**Pan2010**	**Pai2011**	**Mar2010**	**Fut2010**	**C2012(1)**	**C2012(2)**	**Pan2012**	**C**	**B**	**E**	**D**	**A**
**Pan2010**	–	0.972	0.991	0.985	0.964	0.971	0.989	0.961	0.961	0.955	0.961	0.961
**Pai2011**		–	0.974	0.973	0.983	0.994	0.976	0.984	0.984	0.978	0.984	0.984
**Mar2010**			–	0.987	0.970	0.975	0.995	0.961	0.961	0.955	0.961	0.961
**Fut2010**				–	0.964	0.974	0.990	0.960	0.960	0.953	0.960	0.960
**C2012(1)**					–	0.985	0.970	0.970	0.970	0.963	0.970	0.970
**C2012(2)**						–	0.978	0.981	0.981	0.974	0.981	0.981
**Pan2012**							–	0.963	0.963	0.957	0.963	0.963
**C**								–	1.000	0.993	1.000	1.000
**B**									–	0.993	1.000	1.000
**E**										–	0.993	0.993
**D**											–	1.000
**A**												–

*Geographic location and year are indicated for control cases (numbers in parentheses indicate multiple cases in the same year; case-patient identification letters (A–E) are given for cases from this study. – indicates alignment of the same sequence.

**Figure 2:**
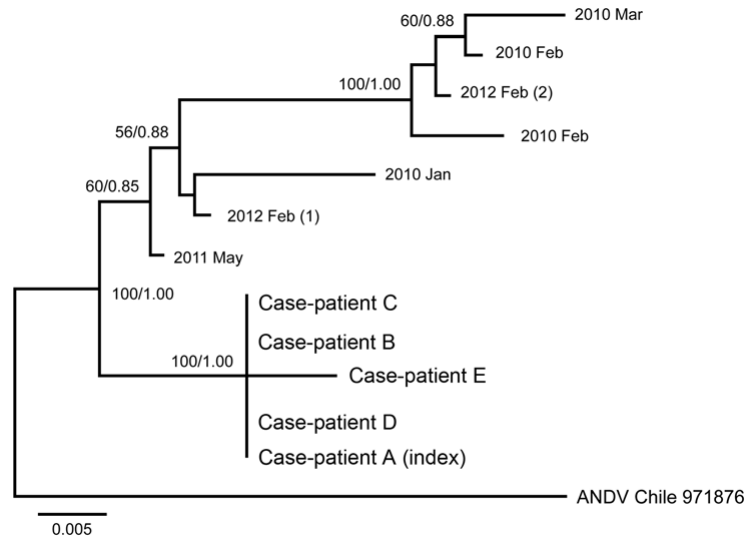
Phylogenetic analyses of the medium RNA segment (Gc and Gn) of concatenated sequences of Andes hantavirus (ANDV). Isolates from the case-patients (A–E) from the 2011 outbreak in Chile were compared with control samples from the same geographic region (indicated by year isolated; number in parentheses indicates multiple isolates from the same year) and an ANDV sequence from GenBank (bottom isolate on tree; accession no. NC_003467.2). Scale bar indicates substitutions per site.

The phylogenetic analyses through ML and Bayesian methods revealed similar topologic results; thus, a single tree is shown (Figure 2). Results show 2 major groups with strong support provided by the bootstrap and posterior probability values. The group of samples that included the Corral cases is clearly separated from other major clustering that includes ANDV sequences from other localities in Chile.

## Discussion

ANDV is the only hantavirus for which person-to-person transmission has been reported (*7*). Our study of a case cluster in Chile provides epidemiologic and molecular evidence that strongly supports the conclusion that 4 of 5 cases resulted from person-to-person transmission of ANDV, including 2 cases of nosocomial transmission.

Most of the reports of person-to-person transmission of ANDV share common traits that constitute potential risk factors for virus spread (*7*–*9*). These features were also observed in this cluster. First, the period of the disease during which the acute case-patient and the household contact or health care personnel have close contact is primarily the febrile prodrome phase, when symptoms are nonspecific for hantavirus. Second, the number of days from exposure to an index case-patient and the onset of symptoms among additional cases ranges from 12 to 27 days (*7*,*26*), consistent with the intervals observed in our report. In the 2 cases for which environmental exposure was reported, the estimated incubation period after that exposure exceeded the longest reported incubation range of 42 days for ANDV (*3*,*11*). In contrast, in these 2 cases the estimated incubation periods from exposure to a case-patient to onset of symptoms was 13–27 days. Finally, the viral genetic characterization established that viruses from the case cluster shared a high nucleotide sequence identity in Gn and Gc fragments, the most variable viral genomic regions (*6*).

During the prodrome, when symptoms are nonspecific, consideration of ANDV infection and early diagnosis might be triggered by a history of environmental exposure (*1*,*2*) or close exposure to another confirmed case-patient within the known incubation period (*3*,*6*). In this cluster, all the cases appeared in a geographic region that is considered an endemic risk area for hantavirus (*26*,*27*). However, no other cases had occurred in this town since 2010, and our epidemiologic and virus sequence analysis showed that the main risk factor for all the 4 additional cases was the patient’s close contact with a symptomatic, HCPS case-patient (*6*,*28*).

One case of nosocomial transmission of the virus has been previously reported in Argentina (*7*), and evidence of this transmission has been sought in Chile (*12*). We document 2 cases of nosocomial transmission of ANDV, from the index case-patient to a nursing assistant and to a housekeeper, even though their contact with the patient was limited to kissing the patient on the cheek and to handling bedding and gowns (no invasive procedures). Two seroprevalence studies performed soon after recognition of hantavirus in Chile did not reveal a higher proportion of antibodies against ANDV among hospital personnel when compared with the general population (*11*,*12*).

In our study, ANDV infection was not diagnosed in the index case-patient until he had been ill for 31 days, which resulted in a wider time frame of exposure for health care personnel. The patient had a history of diabetes mellitus but no history of any other immunodeficiency that might explain his initial negative serologic test. However, the initial testing was not repeated, so we cannot rule out the possibility of a false-negative result.

For case-patient C, the clinical manifestation of illness was unusual because she lacked respiratory symptoms and showed meningeal irritability as the main sign of the infection. Viral RNA and specific antibodies were not detected, but a slight elevation in the CSF white blood count and protein level were seen. It is possible that viral RNA was present before CSF testing or that it was below the level of detection by RT-PCR, but the timing of her symptoms is probably inconsistent with a postinfectious process.

It is not clear why person-to-person transmission has been documented for ANDV but not for other hantaviruses. Risk factors associated with close contact, including sexual contact, deep kissing, or sleeping in the same bed or room, have been identified in a prospective study of household contacts of index case-patients with HCPS (*9*). As such, respiratory secretions, saliva, or both may be involved in transmission. Puumala virus RNA has been detected by RT-PCR but not by cell culture in saliva from patients who had hemorrhagic fever with renal syndrome (*29*). The antiviral activity of different human saliva concentrations has been experimentally tested against Hantaan virus, Puumala virus, and ANDV; ANDV was least sensitive to the antiviral effect of saliva (*30*). RT-PCR testing has found ANDV RNA in previous and ongoing studies in blood and in body fluids, including gingival crevicular fluid, saliva, endotracheal fluid, and urine (*31*). ANDV was isolated from blood obtained from a child in Chile before the onset of symptoms or development of ANDV antibodies (*32*), and studies are ongoing to determine which, if any, of the body fluids positive by RT-PCR also contain infectious virus.

To characterize and compare the outbreak viral sequences, we used as reference material a selection of sequences from strains obtained 2 or 3 years earlier in the same ecogeographic region near Corral. All 5 medium fragments obtained from case-patient isolates in this cluster were highly similar to each other but were more distantly related to the reference sequences. The strong relatedness of the viruses in the Corral cluster is supported by high bootstrap and posterior probability values in the phylogenetic analyses. Furthermore, the small segment showed 100% identity between the 5 sequences in this cluster. The dates of exposure to high-risk environments or to persons with ANDV infection, known incubation periods, and 100% sequence identify all support a conclusion of person-to-person transmission (*7*). Our data showed 99%–100% identity for a fragment of 913 bp of the medium segment, supporting identity using different sequences. However, we did not include noncoding region fragments, which might provide additional confirmation of identity.

Our study documents a small but definite risk of nosocomial acquisition of ANDV infection for personnel who care for patients, including handling of bedding and gowns. After this investigation, the Ministry of Health of Chile has recommended, in addition to strict adherence to universal precautions, the use of droplet precautions when ANDV infection is suspected. Use of N95 respirator masks, designed to prevent the inhalation of airborne particles, is recommended for those procedures associated with aerosolization of viral contaminated secretions (e.g., respiratory, saliva) when procedures such as suction or intubation are performed. However, this recommendation should be extended to all personnel who have any kind of direct contact with patients or body fluids, including bedding and gowns.

Finally, all close household contacts and health care personnel exposed to a confirmed ANDV case-patient should be closely monitored for signs and symptoms of infection, such as fever, myalgia, headache, and abdominal pain, during the entire documented incubation period of 42 days, even though in person-to-person transmission of ANDV, the onset of symptoms has usually occurred 12–27 days after close contact with a sick patient (*6*,*9*). ANDV RT-PCR should be performed in addition to testing for specific IgM in any exposed contact in whom fever develops within the incubation period, particularly if testing is done within a few days of the onset of fever and before onset of the cardiopulmonary phase. Results of ANDV RT-PCR on blood cells may be positive as early as 5–15 days before onset of symptoms or detection of ANDV antibody (*9*). As we have documented, RT-PCR can detect ANDV RNA in the rare, symptomatic patient in whom seroconversion is delayed.
